# Intelligent Bio-Impedance System for Personalized Continuous Blood Pressure Measurement

**DOI:** 10.3390/bios12030150

**Published:** 2022-02-28

**Authors:** Ting-Wei Wang, Jhen-Yang Syu, Hsiao-Wei Chu, Yen-Ling Sung, Lin Chou, Endian Escott, Olivia Escott, Ting-Tse Lin, Shien-Fong Lin

**Affiliations:** 1 Department of Medical Engineering, California Institute of Technology, Pasadena, CA 91125, USA; twwang2@caltech.edu; 2 Department of Electrical Engineering, California Institute of Technology, Pasadena, CA 91125, USA; 3 Institute of Biomedical Engineering, National Yang Ming Chiao Tung University, Hsinchu 30010, Taiwan; ericsyu.cm06g@nctu.edu.tw (J.-Y.S.); eric11tw.bt03@nctu.edu.tw (H.-W.C.); yen-ling.sung@cshs.org (Y.-L.S.); choulin.arete09@nctu.edu.tw (L.C.); 4 Department of Cardiology, Smidt Heart Institute, Cedars-Sinai Medical Center, Los Angeles, CA 90048, USA; 5 Division of Cardiology, Department of Internal Medicine, National Taiwan University Hospital Hsinchu Branch, Hsinchu 300195, Taiwan; 6 Cardiovascular Center, National Taiwan University Hospital Hsinchu Branch, Hsinchu 300195, Taiwan; 7 Department of Electrical Engineering and Computer Science, Florida Atlantic University, Boca Raton, FL 33431, USA; eescott2018@fau.edu (E.E.); oescott2020@fau.edu (O.E.); 8 College of Medicine, National Taiwan University, Taipei 10617, Taiwan; 9 Division of Cardiology, Department of Internal Medicine, National Taiwan University Hospital, Taipei 10025, Taiwan

**Keywords:** artificial intelligence, bio-impedance measurement, continuous blood pressure measurement, impedance plethysmography, intelligent system

## Abstract

Continuous blood pressure (BP) measurement is crucial for long-term cardiovascular monitoring, especially for prompt hypertension detection. However, most of the continuous BP measurements rely on the pulse transit time (PTT) from multiple-channel physiological acquisition systems that impede wearable applications. Recently, wearable and smart health electronics have become significant for next-generation personalized healthcare progress. This study proposes an intelligent single-channel bio-impedance system for personalized BP monitoring. Compared to the PTT-based methods, the proposed sensing configuration greatly reduces the hardware complexity, which is beneficial for wearable applications. Most of all, the proposed system can extract the significant BP features hidden from the measured bio-impedance signals by an ultra-lightweight AI algorithm, implemented to further establish a tailored BP model for personalized healthcare. In the human trial, the proposed system demonstrates the BP accuracy in terms of the mean error (ME) and the mean absolute error (MAE) within 1.7 ± 3.4 mmHg and 2.7 ± 2.6 mmHg, respectively, which agrees with the criteria of the Association for the Advancement of Medical Instrumentation (AAMI). In conclusion, this work presents a proof-of-concept for an AI-based single-channel bio-impedance BP system. The new wearable smart system is expected to accelerate the artificial intelligence of things (AIoT) technology for personalized BP healthcare in the future.

## 1. Introduction

Blood pressure (BP) monitoring is an important physiological index for cardiovascular health identification [1,2,3]. The cuff-based digital electronic sphygmomanometer is a non-invasive gold standard to detect BP values. However, the device only provides one-shot systolic BP (SBP) and diastolic BP (DBP) measurements that could inconvenience the users when they are monitoring their health conditions in real-time, especially in hypertension patients. Most of all, post-treatment hypertension patients usually need to modify the medicine dosage based on their recovery conditions. For the risk management of these patients, the long-term continuous BP condition recording is important for extracting the significant symptoms and providing an accurate treatment under a narrow therapeutic window for medical doctors [4]. Among the existing clinical techniques for continuous BP measurement, an arterial cannula is a common approach in clinical applications. However, the measurement procedure is an invasive method that could induce potential risks and complications to the patient [5]. To solve this clinical unmet need, the cuffless continuous BP measurement technique is an efficient approach for long-term cardiovascular healthcare. Most of the technique relies on pulse transit time (PTT)-based methods for cuffless continuous BP measurement, according to the Bramwell–Hill equation. The PTT refers to the propagation time of the pressure pulse wave between the two measurement locations by two physiological acquisition systems, such as double photoplethysmography (PPG) devices [6], and a combination of PPG and electrocardiography (ECG) sensors [7]. However, the multiple physiological devices could impede wearable applications.

Recently, artificial intelligence (AI) is rapidly evolving for clinical classification and prediction tasks that are helpful for big data analyses for long-term physiological monitoring, thus providing accurate therapy strategies that can be referenced by physicians [8,9,10]. For the AI in the cuffless BP studies, some groups establish the deep learning model between the physiological signals and arterial BP (ABP) waveforms from the existing databases. For example, Khalid et al. [11] provided the single-channel PPG-based cuffless BP estimation model that involved the two databases from the Queensland [12] and the multiparameter intelligent monitoring in intensive care II (MIMIC-II) datasets [13] and satisfied the Association for the Advancement of Medical Instrumentation (AAMI) standard criteria. El-Hajj et al. [14] proposed recurrent neural networks (RNN) to establish the correlation between the PPG and BP signals from the MIMIC-II datasets. Li et al. [15] provided a long-short-term memory (LSTM)-based deep learning model using the ECG and PPG signals from the MIMIC-II datasets for a real-time cuffless BP estimation.

Although the aforementioned dataset-based approaches presented the qualified BP accuracy within the AAMI criteria, some limitations exist in practical applications. First, the measurement devices with different specifications exist due to the differences in measured signal morphology that may not be suitable for the developed AI-based BP model in previous studies. Second, the AI-based regression models were only adaptive for the patients in the existing datasets, such as the MIMIC-II, restricting the users that are not included in the datasets from conducting this proposed BP model. Third, personalized health behaviors affect BP, including lifestyle habits, personal information, and the environment [16]. Nowadays, the individual AI-based BP model becomes important in the future implications of BP management, with an eye towards personalized medicine [17,18]. Personalized BP healthcare greatly impacts the development of precise therapy, thus providing better BP control and treatment compliance [19,20]. In consumer health electronics, wearable and smart functions in devices play an important role in personalized healthcare [21,22,23].

To this end, this study aims to develop a wearable intelligent bio-impedance system for personalized continuous BP monitoring. The single-channel impedance plethysmography (IPG) signal acquisition device was implemented to measure real-time bio-impedance signals from the pulsation of the carotid artery. Moreover, the proposed system performs personalized BP model computation by an AI-based algorithm to extract the BP features hidden from the IPG waveforms, and to further achieve personalized BP healthcare. The rest of this paper is organized as follows. Section 2 introduces the physiological correlations between IPG signals and BP, as well as the proposed system design and the ethics statement. In Section 3, the experiment results for the IPG signal measurement and the BP accuracy of the proposed system are presented. The discussion and conclusion are provided in Section 4 and Section 5.

## 2. Materials and Methods

### 2.1. Physiological Correlation between IPG and BP

**Figure 1:**
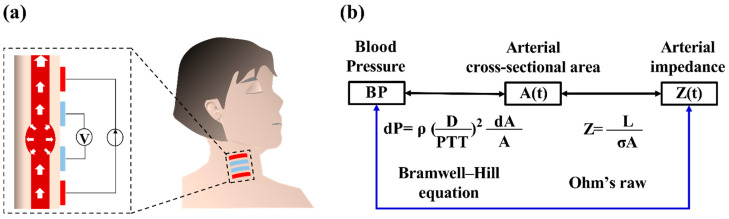
(**a**) Schematic of the IPG technique for hemodynamic measurement. (**b**) Physiological correlation between arterial impedance and blood pressure.

IPG is a bio-impedance technique that is commonly applied in noninvasive physiological measurements [24]. The IPG technique is based on the electric impedance measurement that applies the alternating current into a local area of the body, and then measures the voltage signal. In hemodynamic studies, an IPG-based measurement can extract arterial impedance induced by a small variation in the blood volume [25,26], as shown in Figure 1a. Based on the Bramwell–Hill equation, as in Equation (1), the BP has a strong correlation with the cross-sectional area of the artery. The *dP*, *ρ*, *D*, *A*, and *dA* denote the BP change, blood density, the distance between two physiological measurement locations of the artery, the arterial cross-area, and the change in the arterial area, respectively:

(1) $$dP=\rho {\left(\frac{D}{PTT}\right)}^{2}\frac{dA}{A}$$

Moreover, the small change in the arterial area can be viewed as the arterial impedance variation [27,28], according to Ohm’s law, as in Equation (2), where Z, L, and σ are the arterial impedance, the length of the measured arterial segment, and the arterial conductivity, respectively:

(2) $$Z=\frac{L}{\sigma A}$$

Thus, the arterial pressure can be estimated from the impedance measurement from IPG signals, as shown in Figure 1b. Wang et al. [29,30] and Huynh et al. [31] utilized the IPG technique to establish the BP estimation model between arterial impedance and pressure by a time–domain analysis. However, the IPG signals in the time–frequency analysis have not been extensively investigated. In this study, the time–frequency analysis-based continuous wavelet transform (CWT) was performed to extract the BP features in the IPG signals. Then, the AI-based regression model was used to establish the personalized model between the IPG based-CWT features and the reference BP from the cuff-based sensor.

### 2.2. Wearable Intelligent BP System Design

**Figure 2:**
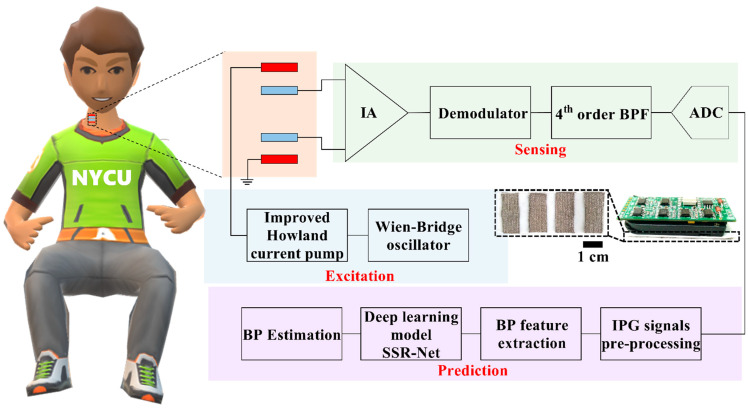
Schematic of the proposed proof-of-concept wearable intelligent bio-impedance system for continuous BP monitoring, including IPG sensing, IPG excitation functions, and AI-based BP estimation.

The wearable intelligent bio-impedance system was implemented to validate the feasibility of the AI-based IPG–BP methodology for personalized medicine. The proposed system can be divided into two main parts, including an IPG sensing device and the AI-based BP estimation, as shown in Figure 2. The IPG sensing device was installed on the subject’s neck for the physiological acquisition from the carotid arterial pulsation, owing to the palpable arterial pulsation and the low BP waveform distortion [30]. The measured IPG signals were transmitted into the proposed AI-based SSR-Net model to further compute the BP information.

#### 2.2.1. IPG Sensing Device

The IPG sensing device consisted of four electrodes, an alternating current source, and a front-end analog circuit. Two pairs of flexible electrodes made with a silver-plated polyester textile with low surface resistivity (<0.05 Ω/inch^2^) were utilized as the electrical function for the current excitation and the physiological sensing. Each rectangular electrode with an area of 2 cm × 0.9 cm and a thickness of 0.03 cm was placed on the carotid artery above the neck as the isometric distribution with a spacing of 0.5 cm.

An alternative current source was implemented by a combination of the Wien–Bridge oscillator and an improved Howland current pump. The sinusoid waveforms, with a frequency of 50 kHz, were produced by the Wen–Bridge oscillator. The voltage-controlled current source (VCCS), improved by the Howland current pump, transforms the signals from a sinusoid voltage to current waveforms with the amplitude and frequency of 0.14 mA and 50 kHz, which follows the human safety guideline [32,33].

To extract the carotid pulse signals in response to the small arterial pulsation, the front-end analog circuit was required to enlarge the small variation in the arterial impedance. The instrumentation amplifier (AD8421, Analog Devices Inc., Norwood, MA, USA) was used to provide an amplification gain of 1000 *v*/*v* and a high common-mode rejection ratio of 110 dB at the input frequency of 50 kHz. The demodulator (AD8310, Analog Devices Inc., Norwood, MA, USA) employs the 50 kHz carrier signal removal from the stage of the instrumentation amplifier. The fourth-order Butterworth bandpass filter, with a narrow bandwidth (0.3–5 Hz), was utilized to cover the typical heart rate ranges from 0.67 Hz to 3.33 Hz [34]. The analog IPG signals were sent into the analog-to-digital converter device (myDAQ, National Instruments, Austin, TX, USA) for further signal processing.

#### 2.2.2. The AI-Based BP Estimation

**Figure 3:**
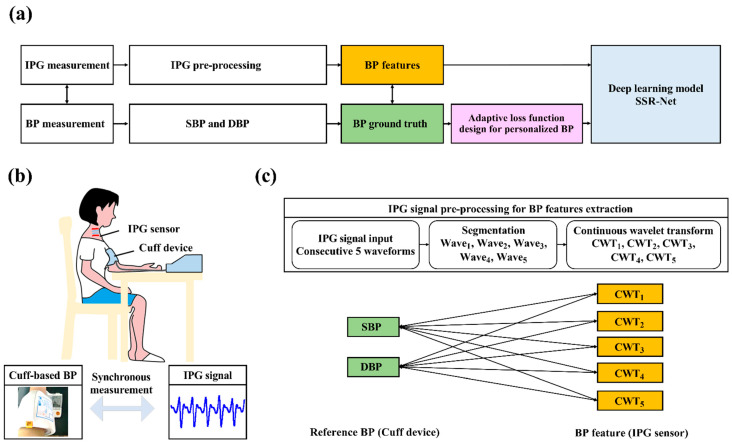
(**a**) Experiment flow chart for physiological acquisition, data pre-processing, and BP estimation. (**b**) Synchronous measurement between the proposed sensor and cuff-based device. (**c**) IPG signals were pre-processed for further BP feature extraction, including signal segmentation and continuous wavelet transform.

In this study, we utilized a deep learning architecture-based SSR-Net model for cuffless continuous BP monitoring. The SSR-Net model is based on the convolutional neural network (CNN) architecture [35]. The SSR-Net model has the merit of a lightweight and complementary two-stream structure [36] that is suitable for real-time monitoring and discrete numerical predictions in BP applications. We also compared the SSR-Net and other lightweight deep learning models, as shown in Table 1. Compared to the MobileNet-V2 and the LSTM approaches for our database training, the SSR-Net provides an ultra-lightweight model size of 213 KB and lower model parameters of 0.04 M, resulting in a CPU interface time of 0.17 s. The experimental flowchart of this study is as follows: IPG signals and cuff-based BP measurements, IPG signal pre-processing for BP feature extraction, dataset arrangement for model training and testing, and a loss function design for the personalized BP monitoring, as shown in Figure 3a.

- IPG Signals and Reference BP Acquisition:

To establish the personalized database for the wearable intelligent bio-impedance BP system, the synchronous measurement for the IPG sensing device and the digital electronic sphygmomanometer (HEM-1000, OMRON, Osaka, Japan) was conducted for 30 measurement trials in the manner shown in Figure 3b. Each trial consisted of SBP and DBP from the cuff-based sensor and five consecutive IPG waveforms from the IPG sensing device, each taking 1 min, due to the 30 s measurement period of the cuff-based BP and the resting of the arteries for 30 s. Thus, the overall acquisition procedure took 30 min for 30 measurement trials in our experiment.

- IPG Signal Pre-processing and BP Feature Extraction:

For each trial, the five consecutive IPG waveforms were segmented into individual waveforms for further feature extraction. The individual IPG signal was converted into a time–frequency analysis by CWT [37]. In this work, the Daubechies 8 (db8) wavelet was used to transform the IPG signals. As shown in Figure 3c, the five pairs of references and features in SBP and DBP were obtained in each measurement trial; thereby, the overall physiological data, with 150 pairs of physiological data for each subject, were acquired.

- Dataset Arrangements for Model Training and Testing:

Overall, 150 pairs of the CWT-IPG based images, and the corresponding reference SBP and DBP values, were categorized into the training and testing datasets. One hundred and twenty pairs of all the datasets were selected for personalized BP model training, and 30 pairs were used to test the BP model accuracy.

**Figure 4:**
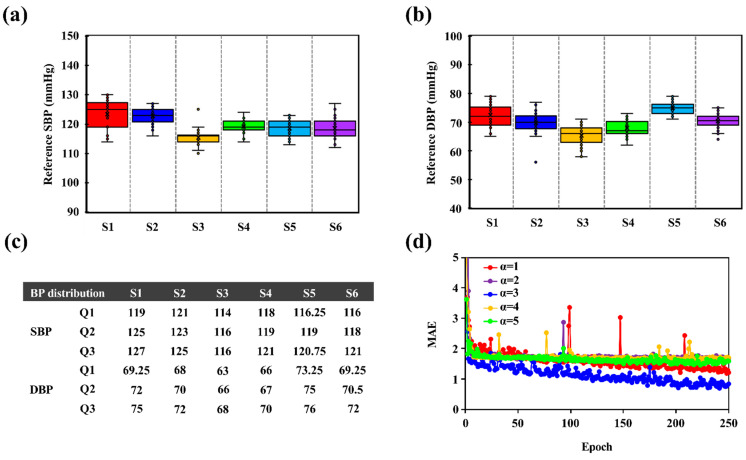
(**a**) Reference SBP and (**b**) DBP distribution for six participants. (**c**) Statistical BP results in quartiles 1, 2, and 3 within six subjects. (**d**) Performance evaluation of model convergence for the penalty terms with different weighting in the interval below quartile 1 and above quartile 3.

- Loss Function Design for Personalized BP Monitoring:

In the process of AI training, the loss function is designed to allow the model to learn the prediction error between the predicted BP by the IPG signal feature (BP_IPG_) and the actual BP by the cuff-based device (BP_cuff_) to further obtain model convergence. In this study, the BP estimation is categorized as a linear regression problem, and it utilizes the mean absolute error (MAE) as the loss function. To obtain the converged personalized BP model during the training stage, the penalty term, in terms of loss function, was modified based on the reference BP distribution of each subject. The penalty term was designed based on being below quartile 1 (Q1) and above quartile 3 (Q3) of the measured reference BP, to accelerate the converge time of the personalized model. Figure 4a–c demonstrates all subjects’ SBP and DBP values, and their quartiles, from the cuff-sensor that was measured 30 times. To evaluate the converge time for the proposed model, the penalty terms with different weighting were implemented, according to Equations (3) and (4). The test results show that the optimal design for the penalty term that was weighted three times in the interval below Q1 and above Q3 obtained a lower epoch to reach the model convergence, as shown in Figure 4d.

(3) $$Loss=|{\;(\mathrm{BP}}_{\mathrm{IPG}}{-\;\mathrm{BP}}_{\mathrm{Cuff}})\times \alpha |,\;{\;\mathrm{BP}}_{\mathrm{Cuff}}<Q1{\;\mathrm{or}\;\mathrm{BP}}_{\mathrm{Cuff}}>Q3\;$$

(4) $$Loss=|{\;\mathrm{BP}}_{\mathrm{IPG}}{-\;\mathrm{BP}}_{\mathrm{Cuff}}|,\;\;Q1<{\mathrm{BP}}_{\mathrm{Cuff}}<Q3$$

- Environment Details:

The Python-based software was used to design and implement deep neural networks. The experiments were performed using Python 3.6.8 inside the Windows 10 Enterprise computer with an Intel^®^ Core™ i7-8700 4.6 GHz processor. Moreover, 64 GB of RAM and a GeForce GTX 1660 Ti 6 GB GPU were equipped on the computer.

#### 2.2.3. Ethics Statement

The human experiment was permitted by the Institutional Review Board of National Yang Ming Chiao Tung University (NCTU-REC-109-012E). A total of six healthy subjects (three males and three females) participated in the experiment, with an age of 24 ± 1 years, a height of 165 ± 8 cm, and a weight of 66 ± 13 kg. The participants consented to participate and provided their written informed approval. During the experiment, they were instructed to remain in a sitting position for the physiological measurement. 

## 3. Results

### 3.1. IPG Signal Measurement and Feature Extraction

**Figure 5:**
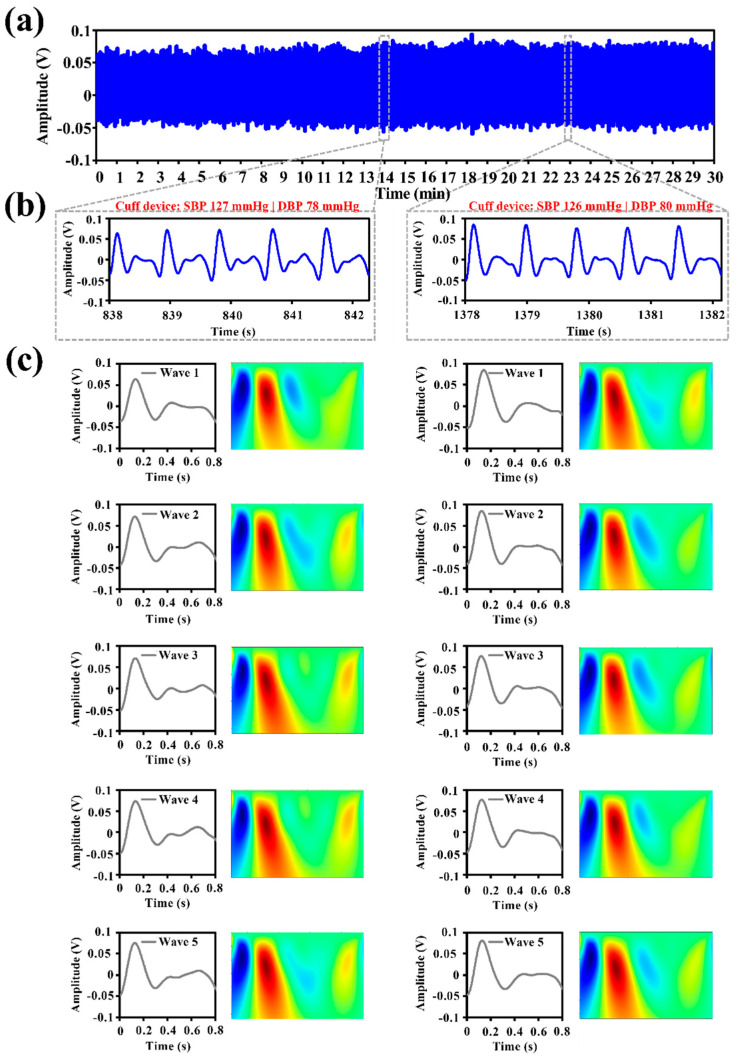
(**a**) IPG signals from a carotid artery for 30 minutes’ measurement. (**b**) Five consecutive IPG waveforms before and after the operating time of the cuff device. (**c**) IPG signal segmentation and further continuous wavelet transform.

The IPG sensing device and digital electronic sphygmomanometer were synchronously conducted for 30 min, as shown in Figure 5a. The consecutive IPG waveforms from the carotid artery above the subject’s neck were measured by the proposed system. To align the measurement procedure of the cuff-based BP device, the five IPG waveforms were selected before and after the operating time of the cuff device, as shown in Figure 5b. In the stage of the BP feature extraction, the IPG signals were divided into a single waveform to further perform the time–frequency analysis by the CWT method, as shown in Figure 5c. 

### 3.2. BP Accuracy Evaluation

**Figure 6:**
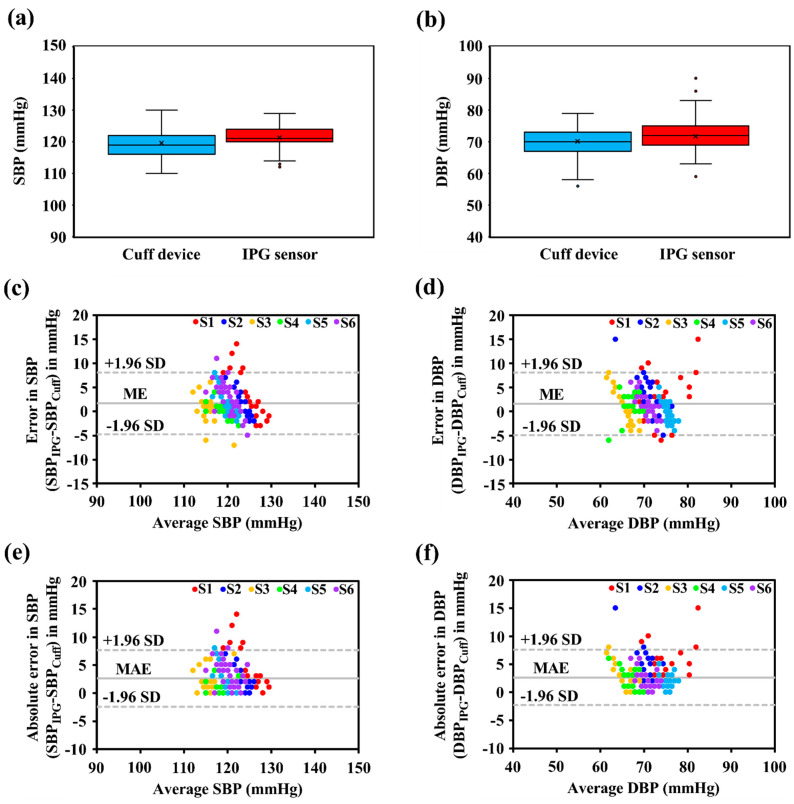
Box plot analysis for subject’s (**a**) SBP and (**b**) DBP distribution from the cuff device and proposed IPG-based system. Bland–Altman plot analysis in terms of ME for (**c**) SBP and (**d**) DBP, respectively. Estimation Error in terms of MAE for (**e**) SBP and (**f**) DBP.

The box plot analysis shows the measured SBP and DBP distributions from the cuff device and the proposed IPG-based system, as shown in Figure 6a,b. The mean SBP from the cuff device (HEM-1000, OMRON) and the IPG-based system were obtained with 119.64 ± 4.32 mmHg (range: 110–130 mmHg) and 121.34 ± 3.61 mmHg (range: 112–129 mmHg); for the DBP, the mean was obtained with 70.13 ± 4.44 mmHg (range: 56–79 mmHg) and 71.69 ± 4.17 mmHg (range: 59–90 mmHg).

To further assess the accuracy performance of the proposed IPG-based system, the statistical results in terms of the mean error (ME) and the MAE, using Bland–Altman plots, were performed as in the evaluation index, according to Equations (5) and (6):

(5) $$\mathrm{ME}=\frac{1}{n}\sum_{i=1}^{n}y_{i}-x_{i}$$

(6) $$\mathrm{MAE}=\frac{1}{n}\sum_{i=1}^{n}|y_{i}-x_{i}|$$

where *x_i_* is the digital electronic sphygmomanometer, *y_i_* is the predicted value of the proposed IPG-based system, and *n* is the number of the testing dataset. 

The SBP accuracy in the testing results indicated that the ME was 1.69 ± 3.28 mmHg (Figure 6c) and the MAE was 2.63 ± 2.58 mmHg (Figure 6e). The ME and MAE were 1.56 ± 3.32 mmHg and 2.66 ± 2.52 mmHg, respectively, for DBP, as shown in Figure 6d,f. Thus, the BP performance of the proposed system satisfied with the standard criteria of the AAMI by less than 5 ± 8 mmHg.

## 4. Discussion

### 4.1. Innovation of Proposed Intelligent Bio-Impedance System

Wearable and intelligent healthcare greatly impacts the development of therapy and management in BP healthcare, especially in personalized medicine [22,38]. The main contribution of this study is to provide a single-channel bio-impedance-based intelligent system with sensing and prediction functions for personalized BP applications.

For the novelty of the sensor configuration design, the proposed single-channel IPG-BP sensor is beneficial for the hardware complexity reduction and for wearable applications, compared to the PTT-based approaches using a multi-channel physiological measurement for BP estimation [31,39,40]. For the novelty of personalized BP healthcare, the proposed intelligent BP system, using an ultra-lightweight AI algorithm, can establish the tailored BP model from the measured signals from each subject. Compared to the AI-based cuffless BP algorithm in existing datasets, such as the MIMIC [11,14,15], the proposed system provides an adaptive BP regression model for each person based on individually measured signals. Such an intelligent BP system design is suitable for personalized healthcare development.

### 4.2. BP Measurement Performance

To evaluate the measurement performance of the proposed wearable intelligent system in BP monitoring applications, the digital electronic sphygmomanometer (as a reference device) was installed for synchronous measurement. The Bland–Altman plot was performed to evaluate the difference in the BP measured by the proposed system and the reference BP by the cuff-based device. Six healthy subjects (three males and three females) with a mean age of 24 ± 1 participated in the human trial. The statistical results of the ME and MAE were utilized as the evaluation metrics to assess the BP accuracy. Based on the statistical results, the accuracy of SBP, in terms of the ME and MAE, was 1.69 ± 3.28 mmHg and 2.63 ± 2.58 mmHg, respectively. The DBP estimation error demonstrated that the ME and MAE were 1.56 ± 3.32 mmHg and 2.66 ± 2.52 mmHg, respectively. The BP performance of the proposed system was satisfied with specifications (less than 5 ± 8 mmHg) based on the standard criteria of the AAMI.

### 4.3. Comparisons with Previous Cuffless BP Works

We compared the proposed IPG-based BP intelligent system with recent studies, as shown in Table 2. Several works relied on multiple physiological parameters to establish the BP model, such as the ECG and the pressure pulse waveform (PPW) [41], two PPG sensors [6], and a combination of the PPG and phonocardiogram (PCG) [42]. Despite the multiple physiological acquisition channels, the approach to providing satisfied BP performance using multi-sensor implementation could increase the complexity of the measurement procedure in practical applications. Recently, several groups attempted to develop deep learning-based BP models using a one-channel physiological signal. For example, Miao et al. [43] proposed deep learning architecture combined with a residual network with LSTM to establish highly accurate BP modeling using the spatial-temporal information of the one-channel ECG signal in the database collected from Fuwai Hospital, Chinese Academy of Medical Sciences. Dal Pont et al. [14] presented an attention-based RNN for cuffless BP measurements using single-channel PPG signals from the MIMIC-II database. Compared to existing dataset-based approaches, we provided a deep learning-based personalized BP model based on the measured signals from an actual single-channel IPG device. The significance of our work is to develop a single-channel wearable sensor combined with an AI-based personalized BP model that is suitable for personalized medicine development.

### 4.4. Limitations and Future Works

Although this work presented an intelligent bio-impedance system for personalized BP monitoring, as well as validating its functional efficacy and BP accuracy, some limitations require further improvement. First, the participants are young healthy people in our experiment. More old-aged subjects and patients with cardiovascular disease will be recruited to make the BP accuracy more reliable in the clinical aspect. Second, the IPG sensing device and the AI-based model will be integrated into a hardware implementation for practical applications. Third, the evaluation of the frequency of calibration will be performed to qualify BP monitoring with changes in measurement conditions, such as an atmospheric humidity-induced skin-electrode impedance change. Fourth, the ambulatory BP measurement technique by the analog front-end and post-processing improvements will be developed for practical applications and daily activities.

## 5. Conclusions

This study develops a proof-of-concept wearable intelligent system for personalized BP healthcare. The proposed system integrates a one-channel IPG sensing device and an AI-based regression model for cuffless continuous BP measurement. Compared to the PTT-based BP device and the MIMIC series dataset-based BP estimation model, our system provides a combined solution with the merits of wearable and intelligent properties in continuous BP measurement. In the accuracy evaluation, the experimental results validated the feasibility of the proposed system, resulting in qualified BP performance. Overall, our work develops a novel BP system to present an insightful view towards next-generation personalized BP healthcare.

## Figures and Tables

**Table 1:** Comparison of different lightweight deep learning models.

Model	SSR-Net	MobileNet-V2	LSTM
Model size	213 KB	13,932 KB	8744 KB
Model parameters	0.04 M	3.50 M	215.99 M
Inference time on CPU	0.17 s	0.29 s	0.25 s

**Table 2:** Comparison of cuffless continuous BP measurement technologies.

**Author**	**Physiological** **Signal**	**Deep** **Learning Model**	**Statistical** **Results**	**BP Estimation Error**	
				**SBP**	**DBP**
Miao et al. [41]	ECG, 2-PPW	-	ME ± SD	1.62 ± 7.76	1.49 ± 5.52
Tabei et al. [6]	2-PPG	-	MAE ± SD	2.07 ± 2.06	2.12 ± 1.85
Marzorati et al. [42]	PPG, PCG	-	ME ± SD	1.47 ± 3.76	0.01 ± 7.55
Miao et al. [43]	ECG	Res-LSTM	ME ± SD	−0.22 ± 5.82	−0.75 ± 5.62
El-Hajj et al. [14]	PPG	Attention based-RNN	ME ± SD	−0.52 ± 4.22	−0.66 ± 2.07
			MAE ± SD	2.58 ± 3.35	1.26 ± 1.63
Our work	IPG	SSR-Net	ME ± SD	1.69 ± 3.28	1.56 ± 3.32
			MAE ± SD	2.63 ± 2.58	2.66 ± 2.52
